# Impact of a non-therapeutic laparotomy in patients with locally advanced pancreatic cancer treated with induction (m)FOLFIRINOX: Trans-Atlantic Pancreatic Surgery (TAPS) Consortium study

**DOI:** 10.1093/bjs/znae033

**Published:** 2024-03-08

**Authors:** Rutger T Theijse, Thomas F Stoop, Quisette P Janssen, Laura R Prakash, Matthew H G Katz, Deesje Doppenberg, Ching-Wei D Tzeng, Alice C Wei, Amer H Zureikat, Bas Groot Koerkamp, Marc G Besselink

**Affiliations:** Department of Surgery, Amsterdam UMC, location University of Amsterdam, Amsterdam, The Netherlands; Cancer Center Amsterdam, Amsterdam, The Netherlands; Department of Surgery, Amsterdam UMC, location University of Amsterdam, Amsterdam, The Netherlands; Cancer Center Amsterdam, Amsterdam, The Netherlands; Division of Surgical Oncology, Department of Surgery, University of Colorado, Aurora, Colorado, USA; Department of Surgery, Erasmus MC Cancer Institute, Rotterdam, The Netherlands; Department of Surgical Oncology, Division of Surgery, The University of Texas MD Anderson Cancer Center, Houston, Texas, USA; Department of Surgical Oncology, Division of Surgery, The University of Texas MD Anderson Cancer Center, Houston, Texas, USA; Department of Surgery, Amsterdam UMC, location University of Amsterdam, Amsterdam, The Netherlands; Cancer Center Amsterdam, Amsterdam, The Netherlands; Department of Surgical Oncology, Division of Surgery, The University of Texas MD Anderson Cancer Center, Houston, Texas, USA; Department of Surgery, Memorial Sloan Kettering Cancer Center, New York, New York, USA; Division of Surgical Oncology, University of Pittsburgh Medical Center, Pittsburgh, Pennsylvania, USA; Department of Surgery, Erasmus MC Cancer Institute, Rotterdam, The Netherlands; Department of Surgery, Amsterdam UMC, location University of Amsterdam, Amsterdam, The Netherlands; Cancer Center Amsterdam, Amsterdam, The Netherlands

## Abstract

**Background:**

Surgery in selected patients with locally advanced pancreatic cancer after induction chemotherapy may have drawbacks related to surgical risks and breaks or delays in oncological treatment, in particular when curative intent resection is not possible (that is non-therapeutic laparotomy). The aim of this study was to assess the incidence and oncological impact of a non-therapeutic laparotomy in patients with locally advanced pancreatic cancer treated with induction (m)FOLFIRINOX chemotherapy.

**Methods:**

This was a retrospective international multicentre study including patients diagnosed with pathology-proven locally advanced pancreatic cancer treated with at least one cycle of (m)FOLFIRINOX (2012–2019). Patients undergoing a non-therapeutic laparotomy (group A) were compared with those not undergoing surgery (group B) and those undergoing resection (group C).

**Results:**

Overall, 663 patients with locally advanced pancreatic cancer were included (67 patients (10.1%) in group A, 425 patients (64.1%) in group B, and 171 patients (25.8%) in group C). A non-therapeutic laparotomy occurred in 28.2% of all explorations (67 of 238), with occult metastases in 30 patients (30 of 67, 44.8%) and a 90-day mortality rate of 3.0% (2 of 67). Administration of palliative therapy (65.9% *versus* 73.1%; *P* = 0.307) and median overall survival (20.4 [95% c.i. 15.9 to 27.3] *versus* 20.2 [95% c.i. 19.1 to 22.7] months; *P* = 0.752) did not differ between group A and group B respectively. The median overall survival in group C was 36.1 (95% c.i. 30.5 to 41.2) months. The 5-year overall survival rates were 11.4%, 8.7%, and 24.7% in group A, group B, and group C, respectively. Compared with group B, non-therapeutic laparotomy (group A) was not associated with reduced overall survival (HR = 0.88 [95% c.i. 0.61 to 1.27]).

**Conclusion:**

More than a quarter of surgically explored patients with locally advanced pancreatic cancer after induction (m)FOLFIRINOX did not undergo a resection. Such non-therapeutic laparotomy does not appear to substantially impact oncological outcomes.

## Teaser

In 28.2% of 663 patients with locally advanced pancreatic cancer, no resection was performed in case of surgical exploration with intention for resection after at least one cycle of (m)FOLFIRINOX. Such non-therapeutic laparotomy had a 90-day mortality rate of 3.0% and similar outcomes with regards to subsequent administration of palliative chemotherapy and overall survival compared with patients who did not undergo surgical exploration.

## Introduction

Approximately 30–40% of patients with pancreatic adenocarcinoma are diagnosed with locally advanced pancreatic cancer (LAPC) because of extensive vascular tumour involvement. In patients with LAPC, upfront surgery is precluded due to high surgical risks and lack of survival benefit.^1,2^ Historically, LAPC is treated with non-curative intent with an overall survival (OS) of approximately 9 months.^2^ The introduction of multi-agent chemotherapeutic regimens, such as (m)FOLFIRINOX (that is a (modified) combination of 5-fluorouracil, leucovorin, oxaliplatin, and irinotecan) and gemcitabine/nab-paclitaxel, has changed this perspective owing to their potential to improve both local and systemic control.^3^ As a consequence, approximately 16–26% of highly selected patients with LAPC in high-volume centres will undergo a resection with curative intent,^4–6^ associated with improved OS.^7^

Accurate response evaluation during and after induction therapy using anatomical, biological, and conditional parameters is important to adequately select patients for surgery.^2^ Unfortunately, conventional contrast-enhanced CT imaging often overestimates the extent of vascular tumour involvement after induction therapy. Nonetheless, several studies reported favourable outcomes after resection of LAPC, despite persistent vascular involvement on imaging.^8–11^ Therefore, international guidelines and societies state not to base response evaluation only on anatomical parameters, as these cannot accurately predict which patients might benefit from surgery.^12,13^ Hence, the 2022 National Comprehensive Cancer Network (NCCN) and 2016 American Society of Clinical Oncology (ASCO) guidelines state that explorative laparotomy may be considered in all patients with LAPC without evidence of metastatic disease after induction therapy, with a significant decrease in carbohydrate antigen 19-9 (CA19-9) levels, and with clinical improvement indicating response to therapy.^12,13^ This more aggressive attitude towards resection, for which radiographic disease response is no longer a prerequisite, results in higher numbers of patients being selected to undergo surgery.

However, this is inevitably accompanied by more patients undergoing an exploration without resection (that is a ‘non-therapeutic laparotomy’), with the associated risks of postoperative complications and prolonged time off systemic treatment, which may lead to disease progression.^14^ Currently, data regarding the impact of a non-therapeutic laparotomy for LAPC treated with (m)FOLFIRINOX induction chemotherapy are lacking, but could guide surgical decision-making. Therefore, the aim of this study was to assess the incidence of non-therapeutic laparotomy in patients with LAPC treated with induction (m)FOLFIRINOX and its impact on short-term mortality, subsequent receipt of chemotherapy, and OS.

## Methods

This study followed the STROBE guidelines.^15^

### Study design and patients

The international Trans-Atlantic Pancreatic Surgery (TAPS) Consortium comprises five high-volume referral centres in the USA (MD Anderson Cancer Center, Houston, TX; University of Pittsburgh Medical Center, Pittsburgh, PA; and Memorial Sloan-Kettering Cancer Center, New York City, NY) and the Netherlands (Erasmus MC Rotterdam; and Amsterdam UMC). All participating centres contributed to the retrospective establishment of the TAPS cohort, including all patients diagnosed with pathology-proven non-metastasized (that is localized) pancreatic adenocarcinoma from 2012 until 2019, who were treated with (m)FOLFIRINOX as initial treatment. Each participating centre obtained ethical approval from local Institutional Review Boards, as well as legal approval for data-sharing agreements.^16^

### Eligibility

For the present study, all patients diagnosed with LAPC were selected. Patients with metastatic disease found at radiological restaging and those with clinical deterioration during induction therapy precluding further treatment were excluded. Therefore, the total study cohort consisted of only patients with LAPC without radiographic signs of metastatic disease at (re)staging and who were fit to potentially undergo surgery. No restrictions were set on duration or type of (m)FOLFIRINOX treatment, switching to second-line systemic chemotherapy or targeted treatments, or subsequent (chemo)radiotherapy.

Patients undergoing a non-therapeutic laparotomy (group A) were compared with patients deemed locally unresectable at restaging who therefore did not undergo surgery at all (group B). A non-therapeutic laparotomy was defined as a laparotomy for which resection was not performed because of intraoperative conditions (that is metastases or local unresectability). However, during non-therapeutic laparotomy, pre-emptive surgery could be performed, including biliary or gastroenteric bypass surgery (or both). Patients undergoing resection were assigned to group C.

### Data collection and definitions

Pre-specified data on patient demographics, details on treatment course, and clinically relevant outcomes were collected by each participating centre and registered in a centralized database. Patient condition was defined according to the Eastern Cooperative Oncology Group (ECOG) performance status.^17^ Detailed information on data collection and anonymization has been described by Janssen *et al*.^16^. Classification of disease stage was based on anatomical criteria applicable at time of diagnosis in accordance with the NCCN guidelines at time of diagnosis or the MDACC classification system (only applicable to patients originating from MD Anderson Cancer Center).^18^ Both systems defined LAPC as arterial encasement and/or inability to reconstruct the portal vein and/or superior mesenteric vein. Full-dose FOLFIRINOX consisted of oxaliplatin (85 mg/m^2^), leucovorin (400 mg/m^2^), irinotecan (180 mg/m^2^), and fluorouracil (2400 mg/m^2^) with/without bolus (400 mg/m^2^) over 46 h every 2 weeks. In case of dose reduction and/or composite changes, the regimen was described as modified (m) FOLFIRINOX. The total number of (m)FOLFIRINOX cycles was defined as all complete cycles (with or without dosage modification) administered until disease progression, surgery, or change in chemotherapeutic regimen. Second-line induction therapy was defined as a switch from (m)FOLFIRINOX to any other chemotherapy regimen due to toxicity or insufficient response. Switch of chemotherapeutic regimen due to disease progression (local or distant) during induction therapy was registered as palliative chemotherapy.

After induction chemo(radio)therapy, patients were restaged and a decision was made whether to proceed to surgery or to continue with palliative therapy. The timing of restaging was dependent on local protocols and expertise, typically varying between four and eight full cycles of induction therapy (including possible switch to second-line chemotherapy). The relative serum CA19-9 response after induction therapy was categorized according to the optimal response cut-off value for LAPC as determined previously.^19^ Patients proceeding to surgery underwent pancreatoduodenectomy, distal pancreatectomy, or total pancreatectomy (defined in accordance with the International Study Group for Pancreatic Surgery).^20^ Adjuvant therapy was defined as the administration of greater than or equal to one cycle of postoperative chemotherapy. Administration of any cancer-directed therapy (for example chemotherapy, immunotherapy, or radiotherapy), in patients who did not undergo surgery and presented with metastatic/recurrent disease after start of induction therapy, was registered as palliative therapy. Administration of any cancer-directed therapy after non-therapeutic laparotomy was also registered as palliative therapy.

### Outcomes

The primary outcome was OS, calculated from the date of pathology-proven diagnosis. Secondary outcomes included 90-day mortality in the surgical cohort and administration of systemic palliative therapy. To assess the impact of occult metastases on patient outcome, a sensitivity analysis was performed. Patients not undergoing surgery and patients undergoing non-therapeutic laparotomy because of occult metastases or unknown reasons were excluded for this sensitivity analysis only.

### Statistical analysis

Data analyses were performed using RStudio: Integrated Development Environment for R (software version 1.3.1093; Boston, MA, USA).^21^ Descriptive statistics were compiled to summarize patient characteristics. Pearson’s chi-squared test or Fisher’s exact test, when appropriate, was used to compare categorical variables and a Mann–Whitney *U* test was used to compare numerical variables. Categorical variables are presented as *n* (%) and numerical variables are presented as median (interquartile range (i.q.r.)). OS was calculated from the date of diagnosis to the date of death or last follow-up and are presented using Kaplan–Meier estimates with corresponding 95% confidence intervals. Patients still alive on the date of final follow-up (31 December 2020) were censored. Median follow-up was calculated through reversed Kaplan–Meier estimates. OS was assessed using the log rank test. Cox proportional hazards regression analysis was performed to correct for confounders that may determine treatment allocation at time of restaging (that is proceed to surgery or continue with chemotherapy). Multiple imputation, used to account for missing data, was based on 10 imputation sets and predictive mean matching using the mice^®^ package. Results were pooled using Rubin’s rules.^22^ Results of the regression analysis are presented as HRs with corresponding 95% confidence intervals. Clinical predictors with *P* < 0.200 in the univariable analysis were included in the multivariable analysis. Backwards stepwise selection was used for the removal of non-significant variables in the multivariable analysis, until all remaining variables were statistically significant. To correct for potential changes over time in LAPC management, a categorical variable was added in the multivariable analysis, distinguishing patients treated during 2012–2015 and patients treated during 2016–2019. To avoid potential interaction between different CA19-9 parameters, only the CA19-9 parameter with the highest statistical significance was tested in the multivariable analysis when multiple CA19-9 variables had *P* < 0.200 in the univariable analysis. The proportional hazard assumption was assessed by visualization of the Schoenfeld residuals and the log(−log(survival)) *versus* log of survival time plot. The proportional hazard assumption was not violated for any of the factors. For all tests, statistical significance was defined as a two-tailed *P* < 0.050.

## Results

**Fig. 1:**
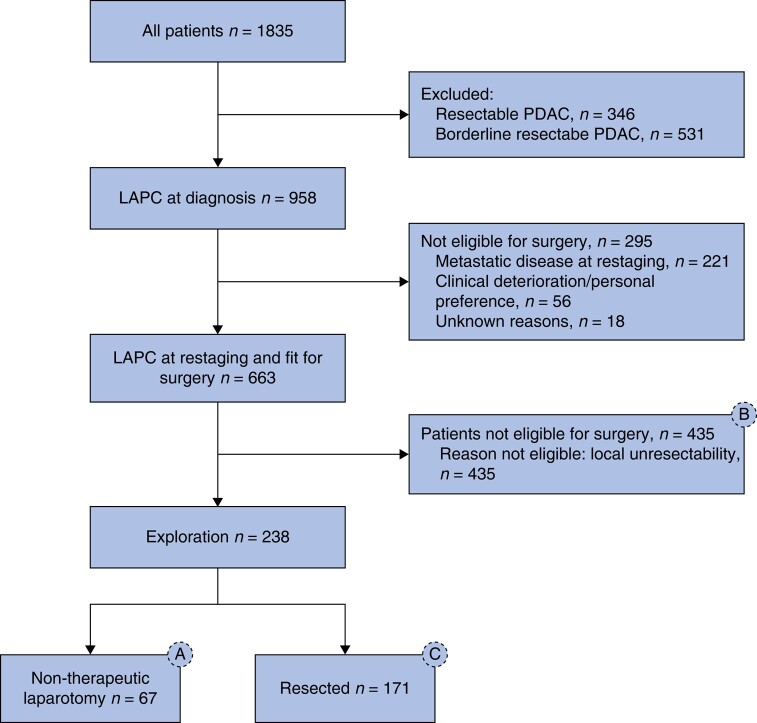
Inclusion flow chart PDAC, pancreatic ductal adenocarcinoma; LAPC, locally advanced pancreatic cancer.

Overall, 1835 patients with LAPC treated with (m)FOLFIRINOX were identified, of whom 958 patients (52.2%) were diagnosed with LAPC at initial presentation. From the 958 patients with LAPC at initial presentation, 238 patients (24.8%) underwent surgical exploration, whereas 221 patients (23.1%) progressed to metastatic disease during induction therapy, 425 patients (44.4%) were deemed unresectable due to extensive local vascular involvement on imaging and/or insufficient tumour marker response, 56 patients (5.8%) refrained from surgery due to personal preferences/clinical deterioration, and 18 patients (1.9%) did not undergo surgical exploration for unknown reasons. After exclusion of all patients with metastatic disease or clinical deterioration at radiological restaging, and all patients who did not proceed to surgery for unknown reasons, the study cohort consisted of 663 selected patients with LAPC who were fit for potential surgery after induction (m)FOLFIRINOX (*Fig. 1*).

### Baseline characteristics of the study cohort

Among the 663 patients, the median age was 63 (i.q.r. 56–68) years and 636 patients (96.7%) had an ECOG performance status of 0 or 1. Induction therapy consisted of only (m)FOLFIRINOX in 585 patients (88.2%). The median number of (m)FOLFIRINOX cycles administered was 8 (i.q.r. 5–9). Subsequently, 438 patients (66.8%) received radiotherapy, of whom 133 patients (30.4%) received stereotactic body radiation therapy (SBRT) and 304 patients (69.6%) received external beam radiation therapy (EBRT). In total, 78 patients (11.8%) were subsequently treated with second-line induction chemotherapy, mostly gemcitabine/nab-paclitaxel (63 of 78, 80.8%). In total, 47 patients (7.0%) received less than four cycles of (m)FOLFIRINOX (one cycle, 14 patients; two cycles, 6 patients; and three cycles, 27 patients). Of these, 13 patients proceeded to surgery who all received second-line gemcitabine/nab-paclitaxel before surgery for a median of 5 months (number of cycles unknown). All patient and treatment characteristics are summarized in *Table 1*.

**Table 1:** Baseline characteristics

Characteristics*	Overall (*n* = 663)	Non-therapeutic laparotomy (group A) (*n* = 67)	No exploration (group B) (*n* = 425)	Resection (group C) (*n* = 171)	*P* (group A *versus* group B)	*P* (group A *versus* group C)
Male	342 (51.6)	44 (65.7)	209 (49.2)	89 (52.0)	0.012†	0.057
Age (years), median (i.q.r.)	63.0 (56.0–68.0)	64.0 (56.0–67.0)	63.5 (57.0–69.0)	61.0 (55.0–66.5)	0.103	0.674
ECOG PS >2	22 (3.3)	1 (1.5)	18 (4.2)	3 (1.8)	0.492	0.999
BMI (kg/m^2^), median (i.q.r.)	25.7 (23.0–28.6)	25.4 (22.2–28.1)	25.7 (22.9–28.7)	25.8 (23.2–28.4)	0.816	0.677
**Location**					0.745	0.570
Head	378 (57.0)	38 (56.7)	250 (58.8)	90 (52.6)	–	–
Body/tail	285 (43.0)	29 (43.3)	175 (41.2)	81 (47.4)	–	–
**Disease characteristics at diagnosis**						
Tumour size (mm), median (i.q.r.)‡	39.0 (31.0–49.0)	39.0 (30.0–47.5)	40.0 (32.0–50.0)	37.0 (30.0–46.3)	0.211	0.891
CA19-9 (U/mL), median (i.q.r.)§	220.3 (47.7–747.0)	221.7 (41.8–636.0)	232.0 (57.5–805.1)	174.3 (28.4–669.2)	0.630	0.575
CEA (ng/mL), median (i.q.r.)§	3.7 (2.2–7.3)	3.9 (2.2–7.0)	3.9 (2.4–7.8)	2.9 (1.8–5.9)	0.756	0.180
**Treatment details**						
Number of cycles of (m)FOLFIRINOX, median (i.q.r.)	8.0 (5.0–9.0)	8.0 (6.0–10.0)	8.0 (5.0–9.0)	7.0 (5.0–8.0)	0.058	0.002†
1–4 cycles	148 (22.3)	11 (16.4)	95 (22.4)	42 (24.6)	0.173	0.001†
5–8 cycles	316 (47.7)	27 (40.3)	194 (45.6)	95 (55.6)	–	–
>8 cycles	199 (30.0)	29 (43.3)	136 (32.0)	34 (19.9)	–	–
Second-line induction therapy	78 (11.8)	4 (6.0)	49 (11.6)	25 (14.6)	0.171	0.067
Type of second-line therapy					0.742	0.484
Gemcitabine/nab-paclitaxel	63 (80.8)	4 (100.0)	43 (87.8)	16 (64.0)	–	–
Gemcitabine/capecitabine	3 (3.8)	0 (0.0)	1 (2.0)	2 (8.0)	–	–
Gemcitabine	6 (7.7)	0 (0.0)	3 (6.1)	3 (12.0)	–	–
Other	6 (7.7)	0 (0.0)	2 (4.1)	4 (16.0)	–	–
Radiotherapy	438 (66.8)	43 (64.2)	305 (72.6)	90 (53.3)	0.155	0.127
SBRT	133 (30.4)	11 (25.6)	89 (29.3)	33 (36.7)	–	–
EBRT	304 (69.6)	32 (74.4)	215 (70.7)	57 (63.3)	–	–
Duration of preoperative therapy (days), median (i.q.r.)¶	231 (189–287)	247 (199–264)	239 (196–303)	202 (162–250)	0.417	0.044†
**Disease characteristics at restaging**						
Tumour size, median (i.q.r.)	31.0 (24.0–40.0)	30.0 (22.5–36.5)	33.0 (25.0–43.8)	26.0 (20.0–34.0)	0.034†	0.160
CA19-9 (U/mL), median (i.q.r.)	44.8 (21.0–147.0)	55.0 (20.0–146.0)	55.0 (25.5–228.4)	33.0 (16.0–74.0)	0.512	0.064
Stable/increase	142 (25.0)	11 (20.0)	105 (28.2)	26 (18.4)	0.108	0.493
<60% reduction	86 (15.1)	5 (9.1)	59 (15.8)	22 (15.6)	–	–
≥60% reduction	341 (59.9)	39 (70.9)	209 (56.0)	93 (66.0)	–	–
CEA (ng/mL), median (i.q.r.)	3.5 (2.2–5.6)	3.4 (2.3–5.1)	3.5 (2.4–5.6)	3.5 (1.8–5.3)	0.437	0.876
RECIST^#^					0.685	0.005†
Progressive disease	39 (10.0)	6 (13.6)	32 (12.8)	1 (1.0)	–	–
Stable disease	210 (53.8)	22 (50.0)	142 (56.8)	46 (47.9)	–	–
Partial response	141 (36.2)	16 (36.4)	76 (30.4)	49 (51.0)	–	–
Complete response	0 (0.0)	–	–	–	–	–

Values are *n* (%) unless otherwise indicated. Pearson’s chi-squared test or Fisher’s exact test, when appropriate, was used to compare categorical variables and a Mann–Whitney *U* test was used to compare numerical variables. *Missing data: age, *n* = 1; ECOG PS, *n* = 5; BMI, *n* = 6; tumour size, *n =* 18; CA19-9 at diagnosis, *n* = 40; CEA at diagnosis, *n* = 247; radiotherapy, *n* = 7; type of radiotherapy, *n* = 1; second-line induction therapy, *n* = 1; size at restaging, *n* = 185; CA19-9 at restaging, *n* = 247; CEA at restaging, *n* = 441; and RECIST, *n* = 273. †Statistically significant. ‡Measured using radiological imaging before the start of induction therapy. §Measured as close to the start of induction therapy as possible. ¶Time from diagnosis to first measurement after total induction therapy was taken as a surrogate marker for duration of preoperative therapy. ^#^Progressive disease defined as 20% increase of largest tumour diameter, partial response defined as 30% decrease of largest tumour diameter, and complete response defined as tumour disappearance. i.q.r., interquartile range; ECOG PS, Eastern Cooperative Oncology Group performance status; CA19-9, carbohydrate antigen 19-9; CEA, carcinoembryonic antigen; SBRT, stereotactic body radiation therapy; EBRT, external beam radiation therapy; RECIST, response evaluation criteria in solid tumours.

### Study groups

Of the 663 patients with LAPC, 238 patients (35.9%) underwent surgical exploration with intention for resection after induction therapy, of whom 67 patients (67 of 663, 10.1%) underwent non-therapeutic laparotomy (group A) and 171 patients (171 of 663, 25.8%) subsequently underwent a resection (group C). The remaining 425 patients (425 of 663, 64.1%) were deemed unresectable due to extensive local vascular involvement on imaging and/or insufficient tumour marker response and therefore did not undergo surgery (group B).

In 28.2% (67 of 238) of all surgical explorations, a non-therapeutic laparotomy was performed. This was due to more extensive vascular involvement than perceived on preoperative imaging in 33 patients (33 of 67, 49.3%) and occult metastatic disease in 30 patients (30 of 67, 44.8%); the reason for non-therapeutic laparotomy was unknown in 4 patients (4 of 67, 5.9%).

### Outcomes

**Fig. 2:**
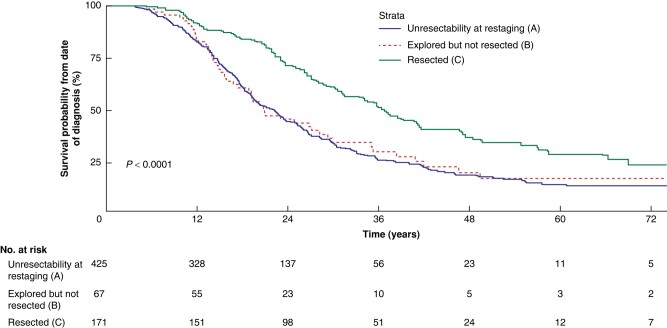
Overall survival

The median follow-up time of all patients was 41.0 (i.q.r. 38.0–47.3) months and 209 patients (31.5%) were alive at the end of follow-up. The median OS was 23.6 (95% c.i. 22.3 to 25.9) months; 20.4 (95% c.i. 15.9 to 27.3) months in group A, 20.2 (95% c.i. 19.1 to 22.7) months in group B, and 36.1 (95% c.i. 30.5 to 41.2) months in group C (*P* < 0.001), as illustrated in *Fig. 2*. The 1-, 3-, and 5- year OS rates were 84.8%, 25.0%, and 11.4% respectively in group A, 82.1%, 21.4%, and 8.7% respectively in group B, and 91.0%, 51.1%, and 24.7% respectively in group C.

The 90-day mortality rate in group A was 3.0% (2 of 67), whereas the 90-day mortality rate in group C was 2.9% (5 of 171) (*P* > 0.999). For patients who did not undergo surgery, 90-day mortality was not calculated, as there was no mutual reference point from which this could be calculated. In group A, 49 patients (73.1%) received palliative therapy, compared with 280 patients (65.9%) in group B (*P* = 0.307). After resection (group C), 75 patients (43.9%) received adjuvant chemotherapy. All outcome measures are summarized in *Table 2*.

**Table 2:** Comparison of outcome measures; total cohort *n* = 663

Outcome measures*	Non-therapeutic laparotomy (group A) (*n* = 67)	No exploration (group B) (*n* = 425)	Resection (group C) (*n* = 171)	*P*†
**90-day mortality**	2 (3.0)	–	5 (2.9)	–
**Palliative/adjuvant therapy‡**	49 (73.1)	280 (65.9)	75 (43.9)	0.307
**Overall survival (months), median (95% c.i.)**	20.4 (15.9,27.3)	20.2 (19.1,22.7)	36.1 (30.5,41.2)	0.752
1-year survival rate, %	84.8	82.1	91.0	–
3-year survival rate, %	25.0	21.4	51.1	–
5-year survival rate, %	11.4	8.7	24.7	–

Values are *n* (%) unless otherwise indicated. *Missing data: palliative therapy, *n* = 22; and adjuvant therapy, *n* = 7. †*P* value for group A compared with group B. ‡Patients in group A and B received systemic palliative therapy and patients in group C received systemic adjuvant therapy.

No difference in median OS was observed between group A and group B (20.4 (95% c.i. 15.9 to 27.3) *versus* 20.2 (95% c.i. 19.1 to 22.7) months respectively; *P* = 0.752). To determine whether non-therapeutic laparotomy was predictive for OS, Cox regression analysis was performed. Non-therapeutic laparotomy was not identified as an independent negative prognostic factor for OS (HR = 0.88 (95% c.i. 0.61 to 1.27)), whereas CA19-9 level at time of restaging was identified as an independent predictor for OS. See *Table 3* for the Cox regression analysis.

**Table 3:** Cox regression analysis—overall survival

Variable*	Univariable analysis		Multivariable analysis	
	HR (95% c.i.)	*P*	HR (95% c.i.)	*P*
**Treatment**				
No exploration	1 (reference)	NA	1 (reference)	NA
Non-therapeutic laparotomy	0.94 (0.69–1.27)	0.681	0.88 (0.61–1.27)	0.486
Resection	0.48 (0.38–0.61)	<0.001	0.45 (0.29–0.70)	0.002†
**Year of surgery**				
2012–2015	1 (reference)	NA	–	–
2016–2019	0.82 (0.63–1.05)	0.120	–	–
**Sex**				
Male	1 (reference)	NA	–	–
Female	0.95 (0.79–1.15)	0.614	–	–
**Age (years)**				
<70	1 (reference)	NA	–	–
≥70	0.97 (0.76–1.22)	0.777	–	–
**ECOG PS**				
0	1 (reference)	NA	–	–
1	1.22 (1.00–1.48)	0.053	–	–
≥2	1.49 (0.93–2.41)	0.102	–	–
**BMI (kg/m^2^)**				
18.5–25.0	1 (reference)	NA	–	–
<18.5	1.47 (0.90–2.42)	0.127	–	–
>25.0	0.93 (0.77–1.12)	0.449	–	–
**Location**				
Head/uncinate process	1 (reference)	NA	–	–
Body/tail	0.86 (0.72–1.04)	0.122	–	–
**Second-line induction chemotherapy**				
No	1 (reference)	NA	–	–
Yes	1.03 (0.78–1.38)	0.817	–	–
**CA19-9 at restaging (U/ml)**				
0–37	1 (reference)	NA	–	NA
>37–100	0.97 (0.69–1.36)	0.860	0.94 (0.68–1.31)	0.735
>100–500	1.79 (1.18–2.69)	0.011	1.79 (1.26–2.52)	0.002†
>500	1.96 (0.81–4.75)	0.173	2.21 (1.39–3.50)	0.003†
**CA19-9 response**				
Stable/increase	1 (reference)	NA	–	NA
<60% reduction	1.20 (0.83–1.73)	0.333	–	–
≥60% reduction	0.74 (0.59–0.93)	0.012†	–	–
**RECIST radiotherapy**				
Stable disease	1 (reference)	NA	–	–
Progressive disease	1.32 (0.77–2.27)	0.328	–	–
Partial response	0.66 (0.50–0.87)	0.006	–	–

^*^Imputed data: age, *n* = 1; BMI, *n* = 6; ECOG PS, *n* = 6; chemotherapy switch, *n* = 1; CA19-9, *n* = 247; and RECIST, *n* = 273. †Statistically significant. NA, not applicable; ECOG PS, Eastern Cooperative Oncology Group performance status; CA19-9, carbohydrate antigen 19-9; RECIST, response evaluation criteria in solid tumours.

In a sensitivity analysis, excluding patients in group B (425 patients) and those patients in group A with metastases (30 patients) or unknown reasons for not undergoing a resection (4 patients), the 90-day mortality rate for the 33 patients undergoing non-therapeutic laparotomy due to more extensive vascular involvement than perceived before surgery was 0% (0 of 33) and the median OS was 25.0 (95% c.i. 15.4 to 40.8) months. The 1-, 3-, and 5- year OS rates for these patients were 87.7%, 35.0%, and 18.0%, respectively.

## Discussion

This international multicentre observational study, on the incidence and clinical impact of a non-therapeutic laparotomy in 663 patients with LAPC treated with (m)FOLFIRINOX induction therapy, found that in 28.2% of all surgical explorations no resection was performed (approximately half (44.8%) due to occult metastases), with a 90-day mortality rate of 3%. Compared with patients with LAPC who did not undergo surgery, outcomes for patients undergoing non-therapeutic laparotomy were similar with regards to the chance of receiving further systemic chemotherapy and OS.

Two single-centre studies have reported non-therapeutic laparotomy rates of 27.6% and 31.6% in patients with LAPC undergoing surgery after induction chemotherapy, which are in line with the findings of the present study (28.2%).^23,24^ However, the impact of a non-therapeutic laparotomy on 90-day mortality and survival was not studied previously. Only a few series have reported on outcomes after non-therapeutic laparotomy in patients with localized pancreatic adenocarcinoma (that is resectable, borderline-resectable, and LAPC), demonstrating an association with poor short- and long-term outcomes.^25,26^ A Dutch retrospective nationwide analysis (2009–2013) reported a 34.8% non-resection rate in patients with localized pancreatic adenocarcinoma, with the vast minority of patients (1.7%) being treated with preoperative chemotherapy.^25^ In that study, the 30-day mortality rate was higher in patients undergoing a non-therapeutic laparotomy compared with those who underwent surgical resection (7.8% *versus* 3.8% respectively).^25^ However, after excluding patients with occult metastatic disease, the 30-day mortality rates were comparable (4.7% *versus* 3.8% respectively). A study from the Italian Association for the Study of the Pancreas (AISP) reported a non-resection rate of 39.9% in 10 936 patients undergoing surgery for pancreatic cancer.^26^ The rate of non-therapeutic laparotomy was inversely related to the hospital volume and declined progressively from 62.5% in very low-volume centres to 24.4% in very high-volume centres. The in-hospital mortality was higher in patients undergoing non-therapeutic laparotomy compared with patients undergoing resection (8.2% *versus* 6.7% respectively; *P* < 0.01).^26^ Nonetheless, both studies did not investigate the impact of surgical exploration on short-term mortality and OS compared with patients who did not undergo surgery. Additionally, these studies included all stages of localized pancreatic adenocarcinoma, with a minority of patients receiving preoperative chemo(radio)therapy, which hampers direct translation of these results to patients with LAPC undergoing conversion surgery after induction therapy.

In the present cohort, the 90-day mortality rate in patients with non-therapeutic laparotomy was comparable to that in patients who underwent a resection (3.0% *versus* 2.9% respectively). After exclusion of patients with intraoperatively detected occult metastases, the 90-day mortality rate even dropped to 0%. Additionally, compared with patients with LAPC who did not proceed to surgery, a non-therapeutic laparotomy was not associated with OS, indicating an acceptable long-term risk profile.

Some clinicians advise being reluctant with regards to exploration, as (futile) surgery results in postoperative treatment delay or even complete omission of palliative chemotherapy.^27,28^ Indeed, a study from the nationwide mandatory Dutch Pancreatic Cancer Audit^29^ reported that 33% of patients undergoing resection for pancreatic adenocarcinoma did not receive adjuvant chemotherapy.^28^ Postoperative surgical complications, particularly postoperative pancreatic fistula and post-pancreatectomy haemorrhage, were the strongest predictors for not receiving adjuvant chemotherapy. In line with this finding, only 44% of patients undergoing resection in the present study received adjuvant chemotherapy. This may be related to the unclear additional value of adjuvant chemotherapy after preoperative (m)FOLFIRINOX, particularly in patients who already received greater than or equal to eight cycles preoperatively.^30^ This, in combination with the 20–30% of patients who do not receive adjuvant chemotherapy because of complications, may explain the relatively high rate of patients who did not receive adjuvant chemotherapy. However, the study by Mackay *et al*.^28^ only included patients undergoing resection, for which these results^28^ are not fully applicable to patients with LAPC undergoing a non-therapeutic laparotomy. Nonetheless, the data from the present study showed similar administration rates of palliative therapy for patients undergoing surgical exploration compared with those not undergoing surgery. Furthermore, especially in the era of multi-agent induction therapy, which hampers the predictive value of CT-based restaging,^10^ careful intraoperative staging is of vital importance to prevent non-therapeutic laparotomy and its consequences. Therefore, the 2022 NCCN guidelines state that diagnostic laparoscopy can be considered before surgical exploration in all patients or just in high-risk patients (for example elevated CA19-9, large primary tumour, or highly symptomatic).^12^ A retrospective analysis including 151 patients with metastatic disease detected either by staging laparoscopy (89 patients) or laparotomy (62 patients) found that patients undergoing laparotomy had significantly worse OS compared with patients in whom laparotomy was avoided due to metastatic disease detected using laparoscopy (222 days *versus* 343 days respectively) and started palliative chemotherapy less quickly.^31^ These findings, and the high rate of occult metastases as a driver for non-therapeutic laparotomy in the present study, underline the importance of adequate (intraoperative) staging and the potential value of diagnostic laparoscopy in patients with LAPC undergoing surgical exploration after induction (m)FOLFIRINOX. This should be further assessed by larger prospective studies. Suker *et al*.^32^ found a 19% rate of metastases during staging laparoscopy before chemotherapy in patients with LAPC to metastatic disease, for whom non-therapeutic laparotomy could be prevented. In a sub-analysis of the PREOPANC trial, van Dongen *et al*.^33^ reported a 10% yield of occult metastatic disease using diagnostic laparoscopy. Most interestingly, patients in whom metastases were detected using laparoscopy had a higher rate of receiving palliative chemotherapy compared with patients in whom metastases were detected during laparotomy (76.9% *versus* 30.0% respectively; *P* = 0.040). Some clinicians already routinely perform a diagnostic laparoscopy in the same surgical session as the intended resection, although some ambiguity remains regarding its use, indications, and timing.^34,35^

The improvement of chemotherapeutic regimens for pancreatic adenocarcinoma raises the question of whether or not surgery still has a prominent role in its treatment. Often, pancreatic adenocarcinoma is referred to as a ‘systemic disease’ due to the high likelihood of disseminated micrometastases at diagnosis,^36,37^ which is illustrated by the high rate of disease recurrence in up to 70.2% of patients with LAPC after resection.^38,39^ Consequently, some have questioned the additional value of surgery in LAPC, when technically achievable, and assigned the prolonged survival to induction therapy.^40^ Importantly, the observed difference in survival between patients undergoing resection and patients not undergoing resection is severely influenced by selection bias. As most clinicians use the ‘ABC’ paradigm for clinical classification and decision-making,^41^ patients with the best anatomical, biological, and conditional response to induction therapy are selected for surgery. Therefore, it is impossible to conclude that the improved survival outcomes can be primarily attributed to surgery.^42^ Differences in OS are partially, and possibly even completely, based on patient selection. Only a randomized controlled trial could completely overcome this bias. Interestingly, when excluding patients with metastases found intraoperatively in the non-therapeutic laparotomy group (group A) the 5-year OS rate was 18.0%, which approaches the 5-year OS rate in patients who underwent resection (24.4%). This might indicate that long-term survival is indeed predominantly determined by a tumour’s biological behaviour, rather than surgical treatment itself. On the other hand, when technical advancements allow for a resection in these patients the OS-rate may have been even further improved. Future research should focus on determining the potential role of surgery in these patients.

It is important to interpret the results of the present study in light of several limitations. First, treatment protocols at restaging differed between centres and over time. For example, the extent to which tumour marker response was used as criterion to proceed to surgery was not standardized, as the optimal approach with regards to tumour marker response has not yet been established. Nonetheless, this does reflect real-world variation in clinical practice. Similarly, the NCCN definitions for LAPC changed slightly over time, mostly regarding coeliac axis involvement in left-sided pancreatic cancers. Although this might have influenced decision-making over time, the impact would have been small. Further, biological and conditional parameters are being incorporated in the resectability assessment of LAPC. Hence, the assessment extends beyond anatomy alone, which reduces the impact of these minor anatomical changes in definition. Second, the number of patients (67 patients) undergoing non-therapeutic laparotomy (group A) was relatively small, which makes it more difficult to draw robust conclusions. This, combined with the low 90-day mortality rate, precluded logistic regression analysis to identify predictors for 90-day mortality. Nevertheless, the large sample size of the TAPS cohort illustrates the rarity of this patient group. Third, no data regarding quality of life after non-therapeutic laparotomy were available. Even though oncological outcomes and longevity appeared similar, the quality of life could not be assessed. Additionally, reasons for a switch to a second line of induction chemotherapy were not registered within the TAPS database. Fourth, the TAPS Consortium comprises only high-volume tertiary referral centres, so the outcomes might be influenced by referral bias and institutional treatment decisions.^43^ Indeed, the patient population studied was highly selected as the participating centres were high-volume tertiary referral centres, so the results may not necessarily be generalizable to all patients with LAPC (for example those not treated at high-volume centres or those receiving systemic therapy other than (m)FOLFIRINOX).

As the treatment of LAPC continues to evolve with more effective induction regimens, tailored approaches, and more advanced surgical techniques,^44,45^ future research should focus on identifying which patients benefit most from surgical resection and should thus be selected for surgery.^2^ As tumour anatomy and its vascular extent on preoperative imaging is insufficient for adequate patient selection, research should focus on the incorporation of biological parameters, such as serum tumour markers,^46–48^ functional imaging modalities,^49,50^ and imaging biomarkers.^51^

## Data Availability

Data from the present study are not openly available. The authors are willing to share the data upon reasonable request.
